# Supplementary figures and images for: An interferon-stimulated long non-coding RNA USP30-AS1 as an immune modulator in influenza A virus infection
Source: PLoS Pathog. 2025 Jan 8;21(1):e1012854. doi: 10.1371/journal.ppat.1012854 (PMC11750089; doi:10.1371/journal.ppat.1012854)

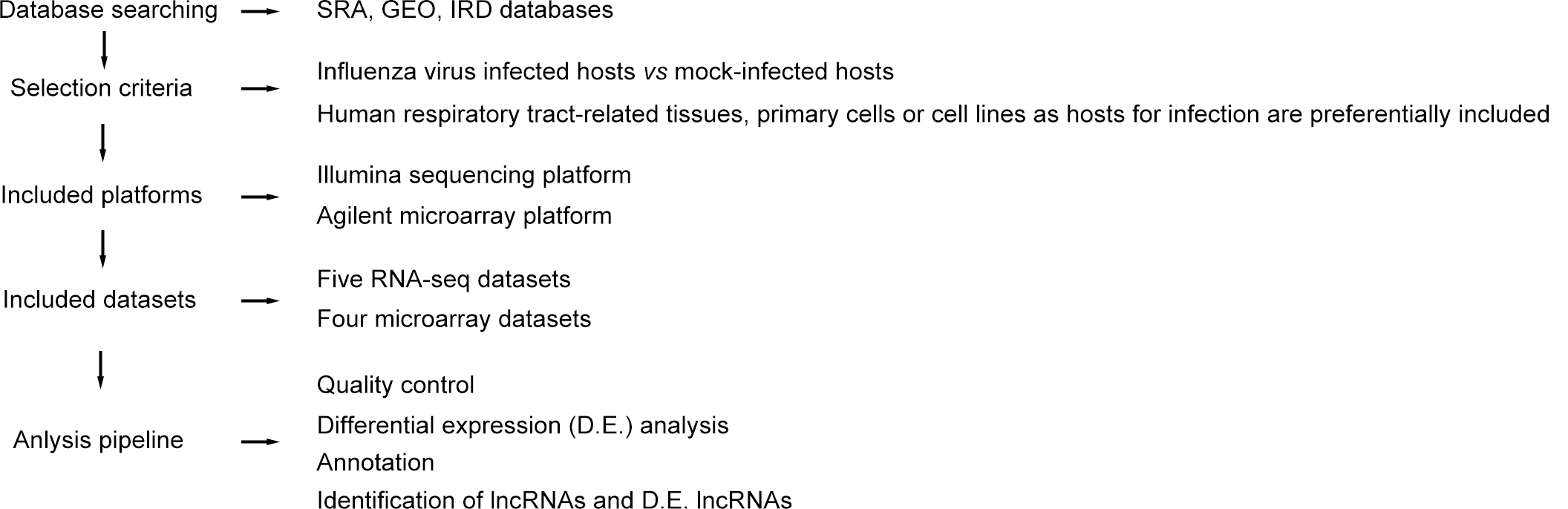

Supplement: S1 Fig — Schematic of working pipeline of database searching, selection criteria, included datasets and downstream analyses. (TIF) [file ppat.1012854.s001.tif]

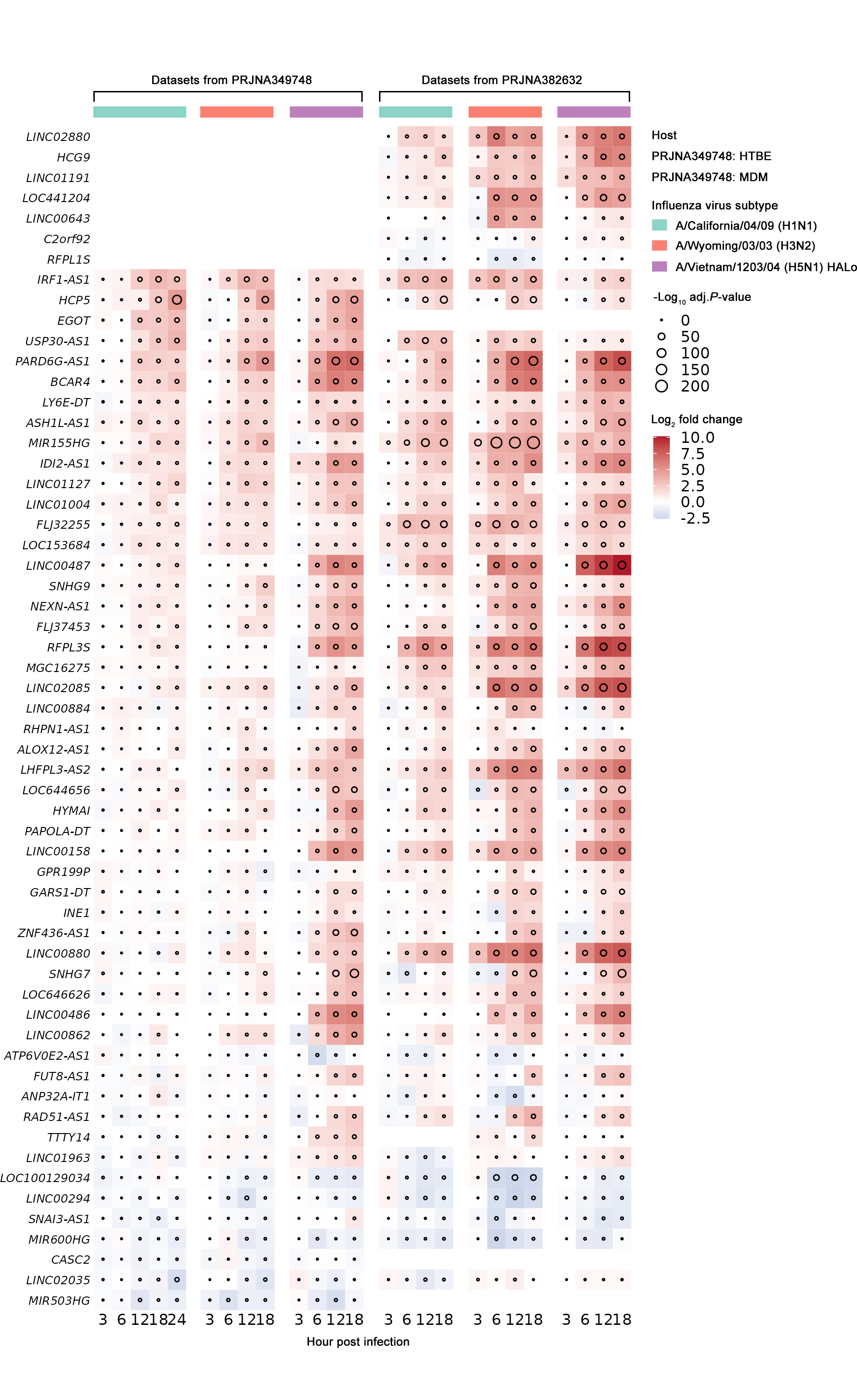

Supplement: S2 Fig — Heatmap showing the dynamic expression of lncRNAs that differentially expressed in infection of at least 5 different IAV subtypes in included bulk RNA-seq datasets. (TIF) [file ppat.1012854.s002.tif]

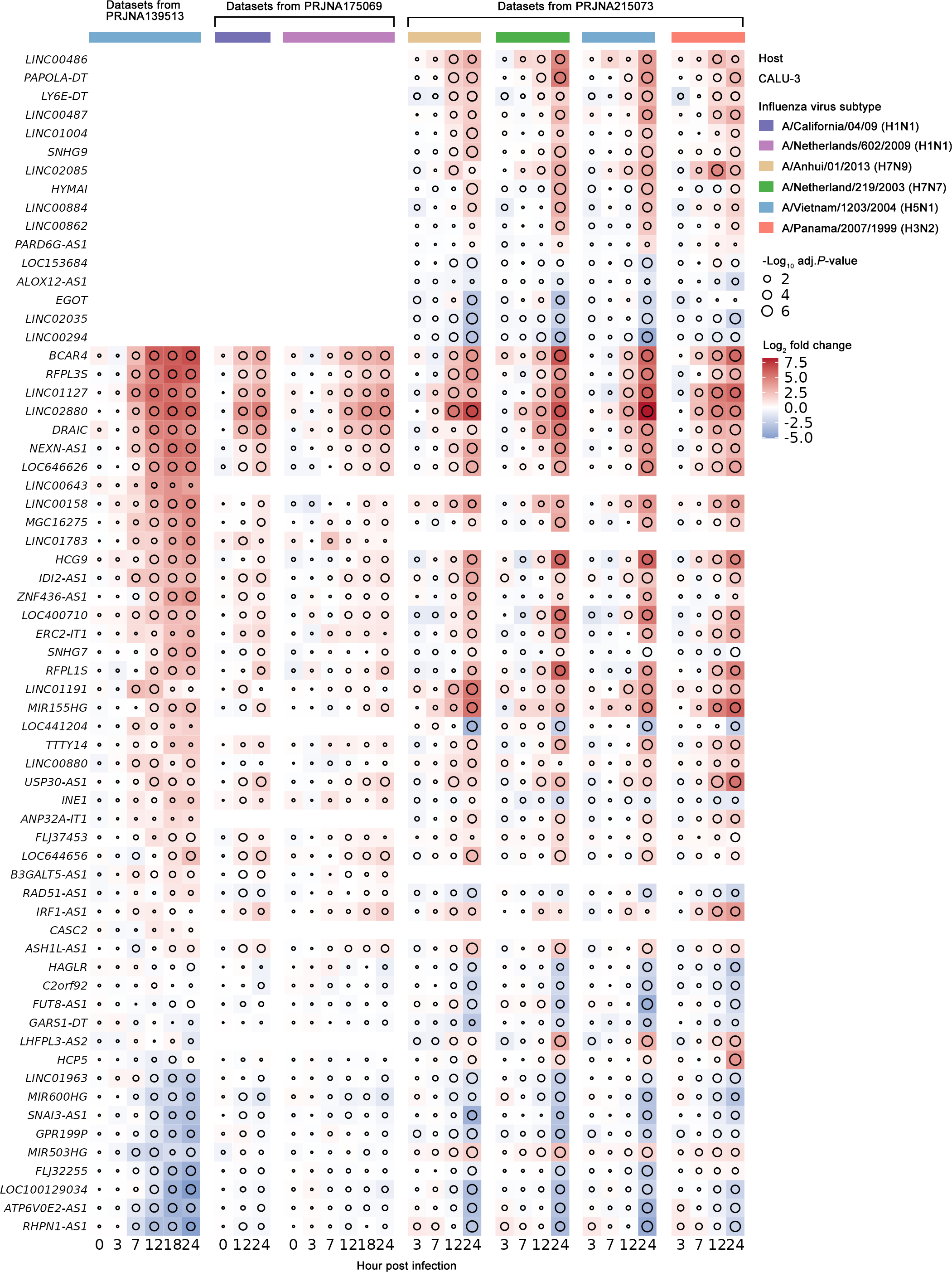

Supplement: S3 Fig — Heatmap showing the dynamic expression of lncRNAs that differentially expressed in infection of at least 5 different IAV subtypes in included microarray datasets (selected example was presented if IAV subtype was included by various included datasets). (TIF) [file ppat.1012854.s003.tif]

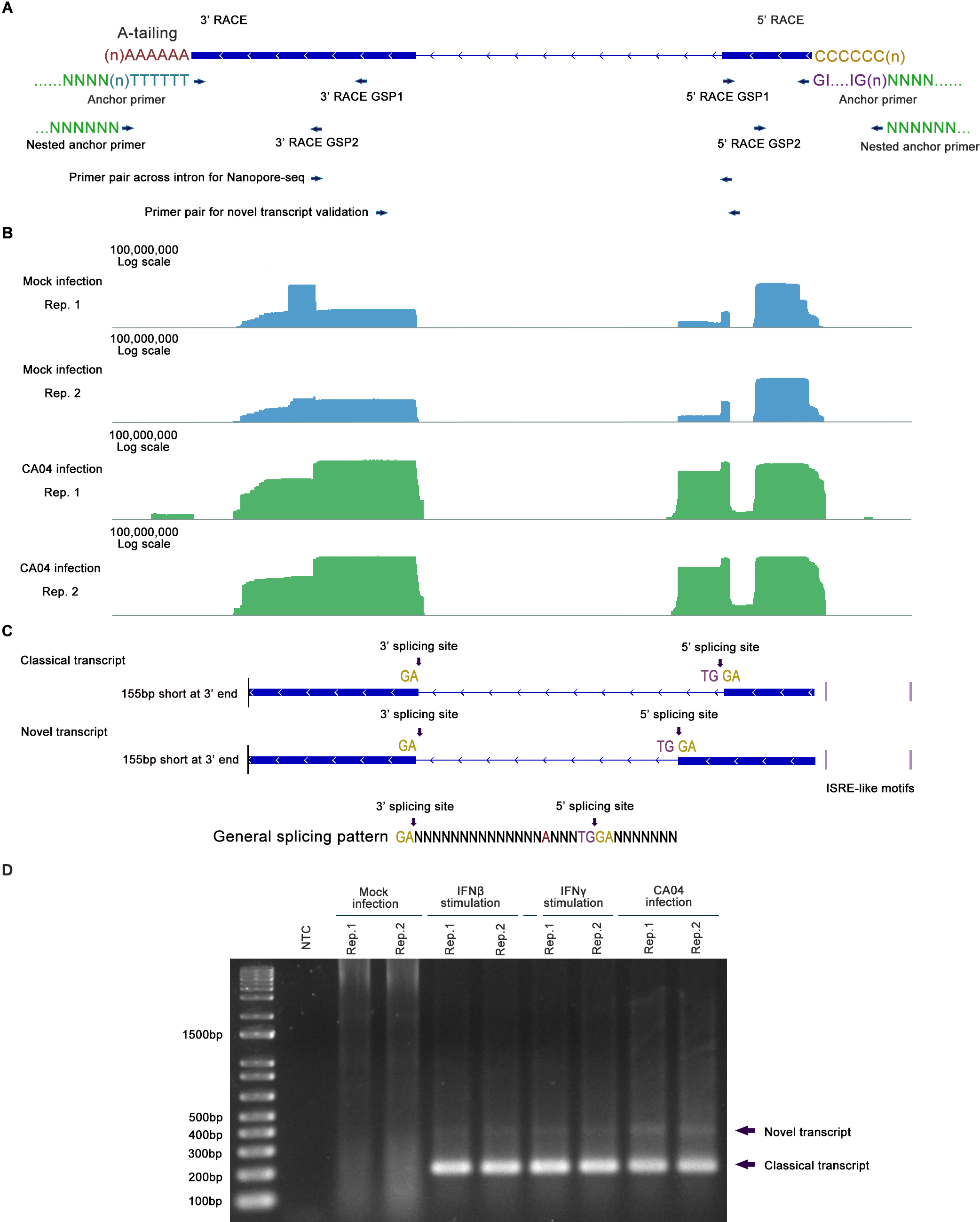

Supplement: S4 Fig — (A) Primers used for USP30-AS1 5’ RACE, 3’ RACE and exon-exon junction PCR, as well as primers for validating the results of exon-exon junction PCR. (B) Coverage (log scale) of mapped reads in USP30-AS1 genome from A/California/04/09 (H1N1) infected A549 cells or PBS mock infected A549 cells in duplicates. (C) Nanopore-RACE-seq determined TSS and TTS of USP30-AS1 with two ISREs in the upstream of USP30-AS1, and the detected novel transcript of USP30-AS1, as well as the possible RNA transcript splicing pattern. (D) Electrophoresis gel showing validated novel transcript of USP30-AS1. (TIF) [file ppat.1012854.s004.tif]

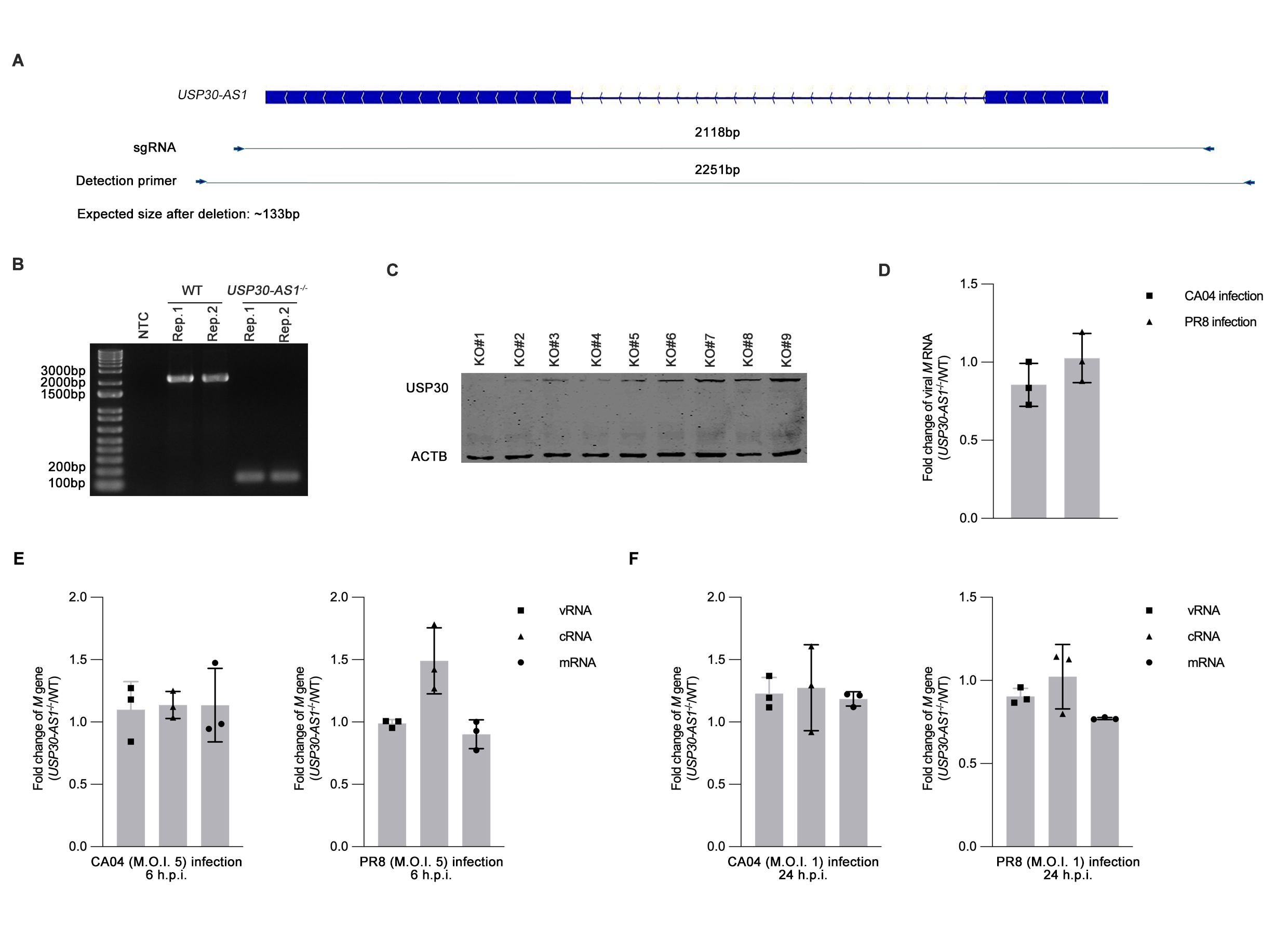

Supplement: S5 Fig — (A) Diagram showing the genomic position of two sgRNAs targeting the upstream and downstream of USP30-AS1 at the same time. (B) Electrophoresis gel showing that compared to 2100 bp PCR product in WT A549 cells, the expected 133 bp PCR product was validated in USP30-AS1 full deletion cells by using detection primer pairs. (C) Immunoblot showing the USP30 protein expression in selected potential USP30 KO cell clones. KO#1 was picked and used for further IAV infection experiments. (D) Detection of expression fold change of IAV viral M gene between either A/California/04/09 (H1N1) or A/Puerto Rico/8/1934 (H1N1) internalized viruses in infected USP30-AS1-/- cells and infected WT cells by qPCR. Student’s t-test was used to test the difference between two groups. Experiment was performed in triplicates. (E) Detection of IAV vRNA, cRNA and mRNA expression fold change of viral M gene between either A/California/04/09 (H1N1) or A/Puerto Rico/8/1934 (H1N1) single cycle infected (M.O.I. of 5) USP30-AS1-/- A549 cells and infected WT A549 cells at 6 hours post-infection (h.p.i.) by qPCR. The bar height represents mean and error bar represents standard deviation. (F) Detection of IAV vRNA, cRNA and mRNA expression fold change of viral M gene between either A/California/04/09 (H1N1) or A/Puerto Rico/8/1934 (H1N1) multiple cycle infected (M.O.I. of 0.1) USP30-AS1-/- A549 cells and infected WT A549 cells at 24 hours post-infection (h.p.i.) by qPCR. Student’s t-test was used to test the difference between two groups. Experiment was performed in triplicates. The bar height represents mean and error bar represents standard deviation. (TIF) [file ppat.1012854.s005.tif]

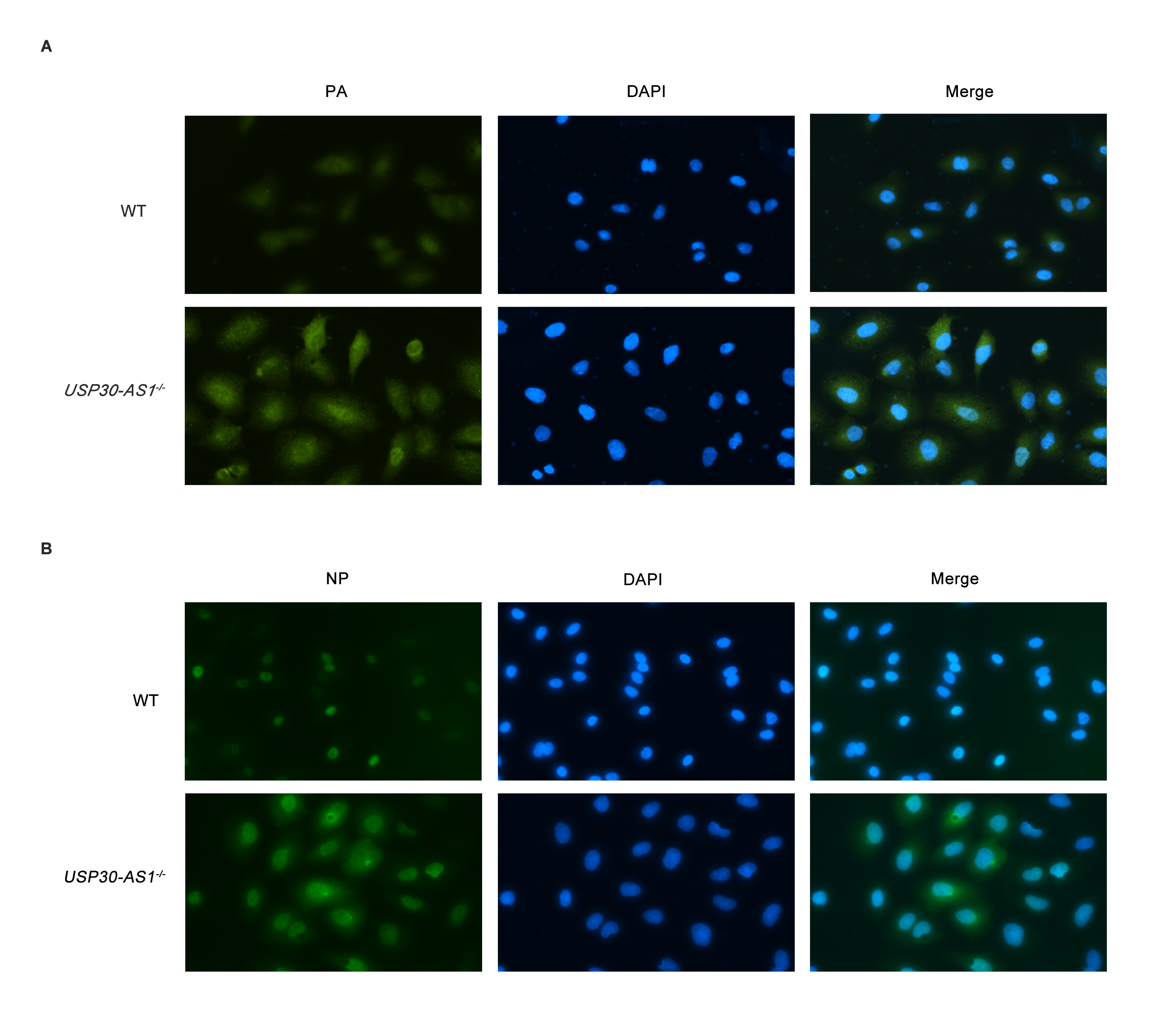

Supplement: S6 Fig — (A) Immunofluorescence of IAV viral protein PA in A/Puerto Rico/8/1934 (H1N1) infected USP30-AS1-/- A549 cells compared to infected A549 WT cells. (B) Immunofluorescence of IAV viral protein NP in A/Puerto Rico/8/1934 (H1N1) infected USP30-AS1-/- A549 cells or infected A549 WT cells. The nuclei were stained by DAPI (4’,6-diamidino-2-phenylindole). Experiment was performed in triplicates. (TIF) [file ppat.1012854.s006.tif]

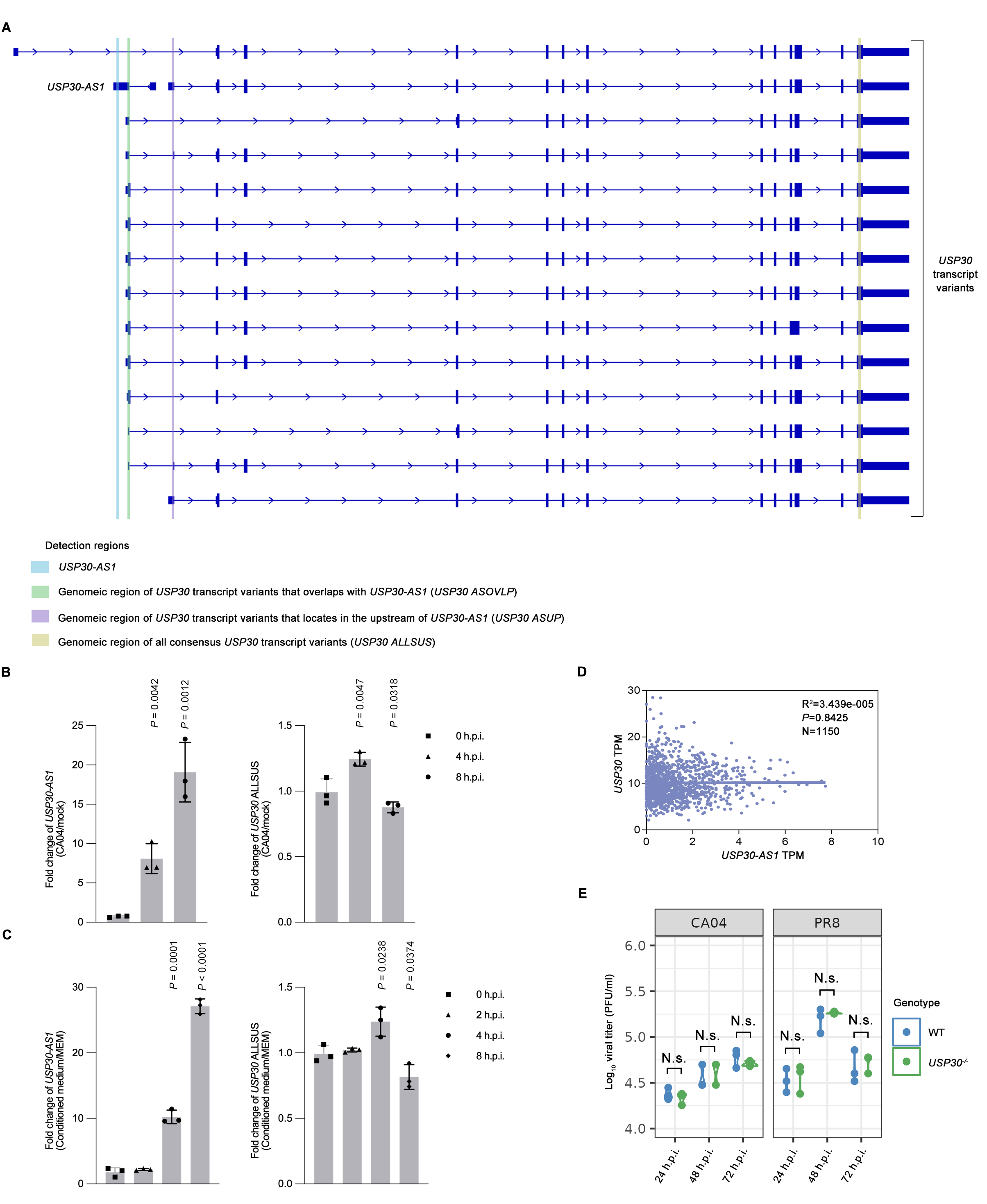

Supplement: S7 Fig — (A) Schematic showing the regions of RNA transcripts of USP30-AS1 and USP30 were detected for gene expression. (B) Detection of the expression fold change of USP30-AS1 (the left panel) and consensus regions of all USP30 transcript variants (the right panel) between single cycle of A/California/04/09 (H1N1) (M.O.I. of 5) infected A549 cells and mock infected A549 cells at 0, 4 and 8 hours post-infection (h.p.i.) by qRT-PCR. Student’s t-test was used to test the difference between two groups in each time point. Significant exact two-sided P-value was reported. Experiment was conducted in triplicates. The bar height represents mean and error bar represents standard deviation. (C) Detection of the expression fold change of USP30-AS1 (the left panel) and consensus regions of all USP30 transcript variants (the right panel) between conditioned medium treated A549 and normal MEM medium treated A549 cells at 0, 2, 4, and 8 hours post treatment by qRT-PCR. Student’s t-test was used to test the difference between two groups in each time point. Experiment was conducted in triplicates. The bar height represents mean and error bar represents standard deviation. (D) The correlation between Transcript Per Million (TPM) of USP30-AS1 and USP30 in 1150 in lung cancer-related tissues. Pearson correlation coefficient was calculated to test correlation coefficient between the two variables. (E) Growth kinetics in the supernatant of influenza virus A/California/04/09 (H1N1) (left) and A/Puerto Rico/8/1934 (H1N1) (right) infected USP30-/- A549 cells compared to infected WT A549 cells. Student’s t-test was used to test the difference between two groups in each time point. Experiment was conducted in triplicates. The bar height represents mean and error bar represents standard deviation. N.s. indicates not significant. (TIF) [file ppat.1012854.s007.tif]

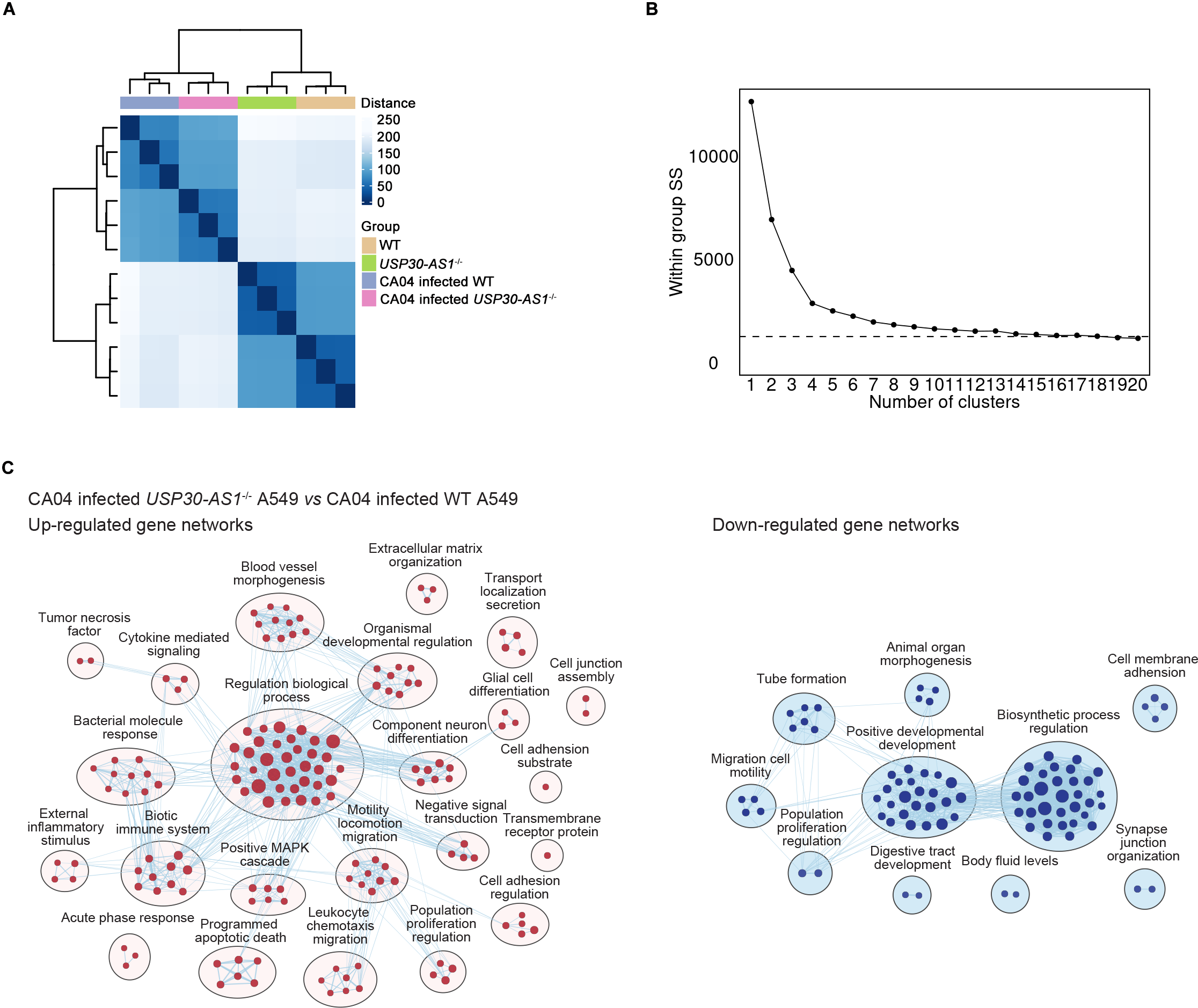

Supplement: S8 Fig — (A) Heatmap showing sample distance matrix across groups. (B) WSS methods to determine cluster number used in K-mean clustering. (C) Enrichment map showing collapsed biological processes networks of GO analysis in up-regulated genes or down-regulated genes in USP30-AS1-/- A549 cells versus WT A549 cells. (TIF) [file ppat.1012854.s008.tif]

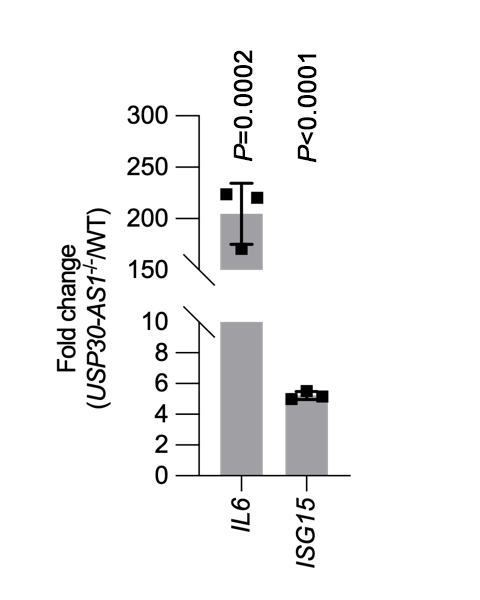

Supplement: S9 Fig — The expression fold change of pro-inflammatory cytokine, represented by IL6, and antivirals, represented by ISG15, between USP30-AS1-/- A549 cells and WT cells in response to 10 μg/ml Poly (I:C) stimulation. Experiment was conducted in triplicates. Student’s t-test was used to test the fold change difference between USP30-AS1-/- and WT group. Significant exact two-sided P-value was reported. Bar height represents mean and error bar represents standard deviation. (TIF) [file ppat.1012854.s009.tif]
