# Supplementary material for: An interferon-stimulated long non-coding RNA USP30-AS1 as an immune modulator in influenza A virus infection
Source: PLoS Pathog. 2025 Jan 8;21(1):e1012854. doi: 10.1371/journal.ppat.1012854 (PMC11750089; doi:10.1371/journal.ppat.1012854)
Supplement: S2 Table — (DOCX) [file ppat.1012854.s011.docx]

Supplementary table 2 Primers used in the study.

| Primers | 5’>3’ |
| --- | --- |
| CA04 *M* gene-F | 5’- ACGGCAAAGGCTATGGAACA-3’ |
| CA04 *M* gene-R | 5’- CACTCCCATTCGCTTCTGGT-3’ |
| PR8 *M* gene-F | 5’-ATGAGYCTTYTAACCGAGGTCGAAACG-3’ |
| PR8 *M* gene-R | 5’-TGGACAAANCGTCTACGCTGCAG-3’ |
| *USP30-AS1*-F | 5’- AATACAGATGGCACACTTCTAG-3’ |
| *USP30-AS1*-R | 5’- GCTCATTAAAGTTCCTTCCTCT-3’ |
| USP30#ALLSUS-F | 5’- GCAATCAGTGGCTGTGGGTCTC-3’ |
| USP30#ALLSUS-R | 5’- CTCTGGTGCTGCATCCTGGAAA-3’ |
| USP30#ASUP-F | 5’- TATTTAATCCTCACAACAGCCCTAT-3’ |
| USP30#ASUP-R | 5’- TCCTGGCTTTGCTATTTCATTT-3’ |
| USP30#ASOVLP-F | 5’- CGGGCCGAGGCGGCGATGA-3’ |
| USP30#ASOVLP-R | 5’- CTGACGGCCGCCCCGGTCCGCA-3’ |
| *ISG15*-F | 5’- CTCTGAGCATCCTGGTGAGGAA-3’ |
| *ISG15*-R | 5’- AAGGTCAGCCAGAACAGGTCGT-3’ |
| *MX1*-F | 5’- GGCTGTTTACCAGACTCCGACA-3’ |
| *MX1*-R | 5’- CACAAAGCCTGGCAGCTCTCTA-3’ |
| *GBP1-*F | 5’-TGACCTACGTCAATGCCATCA-3’ |
| *GBP1-*R | 5’-GCCTCTCTCTCACTGTCCCTGT-3’ |
| *GBP2-*F | 5’-TAAAGGAGGAAGAGCTGAACC-3’ |
| *GBP2-*R | 5’-GGAATGCCACCTGAAAGAGT-3’ |
| *IL6-*F | 5'-AGACAGCCACTCACCTCTTCAG-3’ |
| *IL6-*R | 5'-TTCTGCCAGTGCCTCTTTGCTG-3’ |
| *TNF-*F | 5’-AGACGCCACATCCCCTGACA-3’ |
| *TNF-*R | 5’-AAGGAGAAGAGGCTGAGGAACAAG-3’ |
| sgRNA-*USP30-AS1*-upstream-KO-F | 5’- CACCGAAATATTAGTGTTTGATTAG-3’ |
| sgRNA-*USP30-AS1*-upstream-KO-R | 5’- AAACCTAATCAAACACTAATATTTC-3’ |
| sgRNA-*USP30-AS1*-downstream-KO-F | 5’- CACCGGTAAGTGGTGAAACAGAGAT-3’ |
| sgRNA-*USP30-AS1*-downstream-KO-R | 5’- AAACATCTCTGTTTCACCACTTACC-3’ |
| *USP30*-AS1-KO-detection-primer-F | 5’- TCACAAAAGTGCCTGGAAAG-3’ |
| *USP30*-AS1-KO-detection-primer-R | 5’- CTCCTGTATATTTCTGTTGGAGAG-3’ |
| sgRNA-*USP30*-KO1-F | 5’-CACCGCAGAGTGCATGTCTCCATGG-3’ |
| sgRNA-*USP30*-KO1-R | 5’-AAACCCATGGAGACATGCACTCTGC-3’ |
| sgRNA-*USP30*-KO2-F | 5’- CACCGAACTAGCAATCAGTGGCTGT-3’ |
| sgRNA-*USP30*-KO2-R | 5’- AAACACAGCCACTGATTGCTAGTTC-3’ |
| 5’ RACE-GSP1 | 5’- GCCTCCCTTCCCCACATC-3’ |
| 5’ RACE-GSP2 | 5’- ATAGCAGAGTTGGGAGGAGAA -3’ |
| 5’ RACE-GSP3 | 5’- CCACTCTCCTCGTGATGTCATTTTGC-3’ |
| 3’ RACE-GSP1 | 5’- ATACGACGGTTCCCGAGACA-3’ |
| 3’ RACE-GSP2 | 5’- AGTTGCCTGGGGCTGCTT-3’ |
| AAP | 5’-GGCCACGCGTCGACTAGTACGGGII  GGGIIGGGIIG-3’ |
| AUAP | 5’-GGCCACGCGTCGACTAGTAC-3’ |
| AP | 5’-GGCCACGCGTCGACTAGTACTTT  TTTTTTTTTTTTTT-3’ |
| Junction amplification primer-F | 5’- GGAGCCTAAGCAAATCTGAAAACC-3’ |
| Junction amplification primer-R | 5’- CTGAAGGGAAGAGTGTCAAGGG-3’ |
| Junction amplification nested primer-F | 5’- GCAACTGAGCCCAGCGTAGC-3’ |
| Junction amplification nested primer-R | 5’- CTTCTCCTCCCAACTCTGCTATG-3’ |
| Junction detection primer-F | 5’- TGGTGGGGCCTGCGGATGAA-3’ |
| Junction detection primer-R | 5’- CGCTGTCTCGGGAACCGTCGTAT-3’ |
| Uni12 | 5’-AGCAAAAGCAGG-3’ |
| Uni13 | 5’-AGTAGAAACAAGG-3’ |
| *ACTB*-F | 5’- GGCGGCACCACCATGTACCCT-3’ |
| *ACTB*-R | 5’- AGGGGCCGGACTCGTCATACT-3’ |
| *GAPDH*-F | 5’- GAGTCAACGGATTTGGTCGT-3’ |
| *GAPDH*-R | 5’- TTGATTTTGGAGGGATCTCG-3’ |
