# Supplementary material for: An interferon-stimulated long non-coding RNA USP30-AS1 as an immune modulator in influenza A virus infection
Source: PLoS Pathog. 2025 Jan 8;21(1):e1012854. doi: 10.1371/journal.ppat.1012854 (PMC11750089; doi:10.1371/journal.ppat.1012854)
Supplement: Attachment — Submitted filename: reply.pdf [file ppat.1012854.s020.pdf]

Point by Point Response

We thank the editor and reviewers for reviewing our manuscript and appreciate their constructive comments. Our revisions and responses to reviewers' suggestions are listed below in detail. We believe that this revised version of the manuscript has been significantly strengthened, and hope reviewers find it helpful for the manuscript acceptance.

Reviewer's Responses to Questions

Part I - Summary

Please use this section to discuss strengths/weaknesses of study, novelty/significance, general execution and scholarship.

Reviewer #1: The manuscript by Cao et al. addresses a relevant and novel aspect in the field of virus-host interactions. It studies the contribution of long non-coding RNAs to the host cell response against influenza infection.

Taking advantage of public RNAseq or microarray datasets (5 RNAseq and 4 microarray datasets) generated from human cells infected with influenza virus, the authors established a pipeline to identify differentially expressed lncRNAs common to infections with different influenza strains. Sixty-five lncRNAs were identified, with two of them being the most universally up regulated in cells infected with eight different influenza strains. Only one of them, USP30-AS1, was selected for further analysis.

Suppression of USP30-AS1 in knock-out (KO) A549 cells led to a significant increase in influenza viral titers, suggesting that USP30-AS1 had antiviral activity. Higher viral titers were associated with an increase in the levels of structural proteins with no significant effects in the amount of viral vRNA, cRNA and mRNA. RNAseq experiments showed an enhanced pro-inflammatory response in the absence of USP30-AS1 expression, suggesting an anti-inflammatory effect during infection with influenza virus.

The experimental results shown in the paper are promising and suggest a contribution of USP30-AS1 to the regulation of the inflammatory response in influenza virus infection. However, there are some concerns that should be addressed.

We thank the reviewer for the overall positive comments.

Reviewer #2: *This is a very interesting study by Cao et al, investigating the expression of long non-coding RNA (lncRNA) in different cell types after influenza virus infection. ……*

Overall, this is an interesting study on the role of lncRNA during influenza virus infection in vitro. The inclusion of the USP30 knockout cell data supports the overall conclusions that USP30-AS1 (and not USP30) inhibits influenza virus replication. Unfortunately, the authors did not assess the cytokine response in these cells to differentiate between potential direct effects of the lncRNA on USP30 expression and effects of USP30-AS1 on the virus and the host cell response. Ideally, the authors would have trans complemented the knockout cells with in vitro transcribed RNA (potentially packaged in LNP) to support their claims. Finally, it is unclear how USP30-AS1 controls protein expression after virus infection and how USP30-AS1 functions inside cells.

We appreciate the reviewer for these very constructive suggestions.
1) Relationship between *USP30-AS1* and *USP30*
We thank the reviewer for bringing up the issue concerning the relationship between *USP30-AS1* and *USP30*. Comparing another most universally differentially expressed lncRNA IRF1-AS1, which had significantly positive correlation of transcriptional activation with nearby protein-coding gene IRF (Johnson, Sargsyan et al. 2024) in LPS stimulation and in colon tissues, no such expression correlation was observed between *USP30-AS1* and *USP30*. We also studied lung cancer-related tissues from the TCGA database (https://www.cancer.gov/ccg/research/genome-sequencing/tcga) and failed to identify a clear relationship between *USP30-AS1* and *USP30* (S7D Fig).

As the studied *USP30* KO cells have no effect on the progeny virus titers, we reason that further characterization of *USP30* might be out of the scope of our study. Nonetheless, we agree that this topic is interesting. In our revised manuscript, we highlight that the relationship between these 2 genes is still undefined (Line 275, track change version) and further investigation between their expressions in other cellular events is warranted (Lines 485-486, track change version).

2) Cytokine responses of *USP30-AS1* KO cells upon infection.
In our original manuscript, we demonstrated that infected *USP30-AS1* KO cell has higher cytokine gene expressions than its control. As suggested by Reviewer 2, we also measured cytokine gene and cytokine protein expressions from the same experiment. Our new results can validate our original findings. We have included this additional work in our revised manuscript (Fig 5, panels D and E).

3) Use of LNP-mRNA
We appreciate the suggestion of using the LNP-mRNA approach for trans complementation in knockout cells. But the amount of work and time for its optimization would inhibit us from including this in our study. The proposed work has few potential technical challenges. Firstly, it is a great approach for delivering protein-coding mRNA, as compared to protein-coding mRNA, lncRNAs have higher requirements of their secondary folding conformation, which can't be always correctly guaranteed in the *in-vitro* transcription system. Secondly, when cellular

machinery processes lncRNAs, together with RNA splicing, many accessory proteins that signals processed lncRNAs export or import to the aimed cellular compartments, which is also lacking in the system. Thirdly, base modification in the RNA level is also not perfectly introduced in the LNP-mRNA system. The last might be, as also one of the major concerns in plasmid-based approach for lncRNAs delivery, exogenous lncRNAs can trigger viral sensor immune response, and which is induced in part because the wrong spatial location of delivered lncRNA as listed above, and in part due to over-delivered lncRNA dose, therefore an optimization of delivery amount might be needed.

Nonetheless, we agree that this is a great suggestion. We have included this as a future experiment in our discussion (Lines 540-542, track change version).

4) Role of *USP30-AS1* in viral protein expression and other cellular events
We appreciate the reviewer for highlighting the question as to how *USP30-AS1* controls the actions of influenced protein in IAV infection. It is a very important question, however, there are multiple possibilities from different regulatory levels, requiring a series of attempts and long-term efforts. It would be a very exciting research direction for future investigation, in which we have added a description about study limitations under the discussion section (Lines 527-534, track-change version).

Major comments:
1. The title of the paper suggests USP30-AS1 is regulating antiviral immunity. What data support this conclusion? Based on the data presented, I see a model whereby the increase in virus protein production in the KO cells support higher levels of virus in the supernatant of infected cells. More virus and viral protein drive a stronger host cell response. An alternative model, based on a publication in Theranostics in 2022, implies that the loss of USP30-AS1 enhances the cellular response to virus infection, which in the case of influenza virus and A549 cells could be pro-viral. Demonstrating which of these two or other models is more likely would enhance the significance of the paper.

1) We acknowledge that a better title might be needed. We have revised the title to fit the general arguments as suggested (Lines 1-2, track-change version).

2) We appreciate this reviewer for proposing potential working models about how *USP30-AS1* influences infection-related responses. To gain clues, we treated *USP30-AS1* knock-out cells with interferon γ, and found that loss of *USP30-AS1* can also trigger high inflammatory reaction in interferon stimulation compared to stimulated WT (Fig 5F), suggesting a potential intrinsic deficiency of controlling pro-inflammatory responses. Further, Poly (I:C) stimulation in *USP30-AS1* knock-out cells can also induce a similar strong inflammatory response in comparison to stimulated WT (S9 Fig), indicating viral recognition of dsRNA without further active viral replication is enough to induce this dysregulated high inflammatory response. Therefore, our data aligns with the second model proposed by Reviewer 2. (i.e. loss of *USP30-AS1* dysregulates cellular inflammation-related response that might be a potential pro-viral response). We have

added this comment and additional data in our revised version (Line 519-525, track-change version; Fig 5F and S9 Fig).

2. What is causing the difference in USP30-AS1 expression between virus infection and interferon stimulation? The variable data presentation (fold-change vs USP30-AS1/GAPDH ratio) makes it perhaps difficult to compare.

1) We thank the reviewer for bringing up this important question. We think a potential explanation would be, upon recognition of viral RNA by multiple sensor machineries, viral sensor signaling is able to directly activate a range of viral and inflammatory genes as an effective primary antiviral response (Akira, Uematsu et al. 2006, Pichlmair and Reis e Sousa 2007, Iwasaki 2012). TF IRF3/7 activated from viral sensor signaling or JAK-STAT signaling might bind to ISREs for inflammatory cytokine gene activation, and *USP30-AS1* carries two untypical ISREs in the promoter region. This also supports the above explanation. Besides, due to the engagement of multiple sensor machinery, inhibition of single transducer proteins in the cascade might be not sufficient to block the full signaling, resulting in downstream activation. We have added a detailed explanation in the results and discussions section (Lines 325-327, Lines 337-340 and Lines 498-509, track-change version).

2) We thanks for the suggestion. In the revised version, all panels in the same figure have been presented in a consistent format as fold-change. We hope this consistency of data presentation will be helpful if reads wants to compare these data further.

Minor comments:
Typo in Figure S5F…infetion.

We have changed the presentation format as suggested. This issue has been fixed (S5E, Fig).

Reviewer #3: *Cao and colleagues investigated that role of long non-coding RNAs (lncRNAs) in influenza A virus infection (IAV). Employing publicly available datasets, they identify lncRNAs generally upregulated in the context of IAV infection. Among these was USP30-AS, which was subjected to detailed analyses. In brief, knock out of USP30-AS1 is shown to augment IAV infection, as determined by analyses of viral titers and expression of viral proteins in infected cells. Furthermore, evidence is provided that USP30-AS1 is upregulated by type I, II and III interferon (IFN) and that USP30-AS1 knock out results in a more pronounced upregulation of proinflammatory genes in the context of IAV infection. The results are of some interest to the field. However, important points remain open:*

We thank the reviewer for the overall positive comments.

Part II – Major Issues: Key Experiments Required for Acceptance
Please use this section to detail the key new experiments or modifications of existing experiments that should be <u>absolutely</u> required to validate study conclusions.

Reviewer #1:
1. To further support the physiological relevance of lnc RNA USP30-AS1, it would be important to provide in vivo data confirming that influenza infection in vivo induces an increase in USP30-AS1.

We appreciate the reviewer for mentioning the important question. Due to differences in organisms or other technical reasons, the *USP30-AS1* transcript has only been found to exist in humans. No attempts have been made yet to identify similar transcripts in mice or other species.

To demonstrate the physiological relevance of lncRNA *USP30-AS1*, we obtained human primary alveolar epithelial cells, and infected these primary cells with multiple IAV subtypes. Consistently, the expression of *USP30-AS1* was remarkably enhanced in infected primary cells compared to mock control, suggesting the robustness of the transcriptional activation of *USP30-AS1* to different subtypes of IAV infection. <u>We have included these new data in our revised manuscript (Fig 1B and Lines 190-194, track-change version).</u>

2. Additional information for the interpretation of Fig. 1 should be provided in the text. Columns labeled with "1" apparently represent one specific lncRNA that is differentially expressed in several independent datasets. Do columns labeled with a different number indicate a set of differentially expressed lncRNAs?

We thank the suggestion. We have added additional interpretation about Fig 1A in both main text and figure legend accordingly (<u>Lines 143-156, and Figure 1 legend, track-change version</u>).

3. Experimental results in Fig.S5 C, which validate experimentally the increased levels of USP30-AS1 in human cells infected with influenza virus, should be shown in the main manuscript, instead of in the supplementary material.

We thank the reviewer for this suggestion. <u>We have moved it to the main figures (Fig 1C, track-change version).</u>

4. The methods for relative quantification of USP30-AS1, USP30 and other ISGs in Fig. 2 should be described more clearly. Was qPCR used to quantify these transcripts? ΔΔCt method for relative quantification typically sets to 1 gene expression of the reference sample. In this case, the expression level in the absence of IFN stimulation would be 1 and the expression levels after! IFN stimulation would provide a fold-change value. The values on the Y-axis, which start with "0" do not clearly show the fold-change in the expression level.

We thank the reviewer for noting the issue in the data presentation. Accordingly, we have revised the figure presenting fold-change based on the ΔΔCt method (Fig 2, revised version).

5. Fig. 3. Viral titers in the absence of USP30-AS1 were determined at low MOI (0.1), which corresponds to a multiple-cycle infection. Since protein and RNA levels were studied both at high and low MOI, viral titers should also be analyzed in a single-cycle infection (MOI 5).

We thank the reviewer for bringing up this question. To address the question, we used PR8 with M.O.I. of 5 to infect cells and harvested infected cells at 6 hours post-infection. As data shown below, there was no difference between infected *USP30-AS1* knock-out cells and infected WT cells, and the overall viral titer in the supernatant is very low, suggesting progeny viruses may just start to be released. Given the potential for residual virus inoculum, this new result may not be very useful. To avoid confusion, we have decided not to include in our revised version.

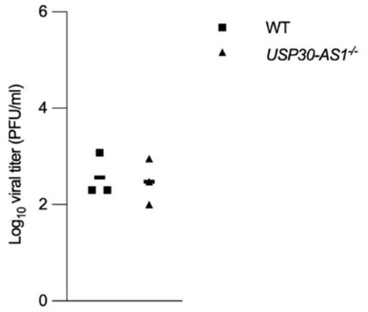

6. The experimental design to determine the relationship between USP30-AS1 expression and the IFN response should be revised. The expression levels of USP30-AS1 are studied in non-infected cells that are treated with IFN (Fig. 4A, B and C). To study whether USP30-AS1 transcription is IFN-dependent, inhibition of IFN signaling pathway with STAT1/ JAK1 inhibitors should be performed in the context of infection. In fact, inhibition of IFN production in infected cells (Fig. E) did not have a significant effect on USP30-AS1 expression. Using cell lines or mutant viruses defective in the IFN response (either IFN production or IFN signaling) might be helpful to determine the physiological relevance of IFN in USP30-AS1 expression.

We appreciate the reviewer for mentioning this important point. To address the question, we performed CA04 infection while keeping JAK-STAT signaling ablated. It showed that CA04 infection in JAK-STAT signaling inhibited cells did not affect the activation of *USP30-AS1* (Fig 4D). Our explanation is other host defense signaling, potentially viral sensor signaling might also play a role in controlling the activation of *USP30-AS1*. This is also supported by literature that viral recognition signaling can directly activate a range of pro-inflammatory genes upon viral RNA detection (Akira, Uematsu et al. 2006, Pichlmair and Reis e Sousa 2007, Iwasaki 2012). We believe results from this new experiment can provide additional insights to guide future work. We have included this into our revised manuscript and commented on the new findings (Fig 4D and Lines 317-340 and Lines 498-509).

We are also thankful for the reviewer's suggestion about using mutant viruses defective in interferon response. As mentioned above, recognition of viral RNA is enough to trigger downstream interferon signaling regardless of any functional deficiency of viruses, therefore it is difficult for us to find such virus strain. Ideally, a cell model with multiple interferon genes and viral sensor genes knock-out would be suitable for testing the purpose, however, it takes a long time to construct the cell model. Nonetheless, we see this as a great idea and suggest this as a future experiment (Lines 540-542, track change version).

7. Fig. 5C. To further support the conclusion that USP30-AS1 contributes to down-regulate the inflammatory response during influenza virus infection, the expression of some relevant genes shown to be differentially expressed by RNAseq analysis should be validated by an alternative technique, such as quantitative PCR.

We have done this as recommended. Consistently, we observed that the expression of these inflammatory genes in CA04 infected *USP30-AS1* knock-out cells were significantly higher than CA04 infected WT cells, suggesting these observations are reproducible. We have included this new data set in our revised version (Fig 5D, Line 393-398, track-change version).

Reviewer #2:

1. Do you see an increase HA and NA expression on the surface of KO cells. This would provide an opportunity to perform more mechanistic and kinetic studies to address the main critique of mechanism and model of this paper.

We thank the reviewer for the constructive suggestion. We have performed suggested experiments with all the HA and NA antibodies we have in our lab. However, stained cells were not able to specific signals in these assays. Therefore, this may not be easy for us to achieve in a short time. <u>Nonetheless, we see this as a great idea and suggest this as a future experiment (Lines 543-545, track change version).</u>

2. Attempt to trans-complement the KO cells with the lncRNA using transfection of mRNA-LNP.

We appreciate the reviewer for the constructive suggestion. As mentioned above (Part 1, Review 2), there are still few concerns even using mRNA-LNP approach for functional intracellular delivery. It also requires a long time to perform the optimization as we haven't constructed a robust experiment system for mRNA-LNP delivering yet. Nonetheless, <u>we believe this is an interesting experiment which warrants to be conducted in future (Lines 540-542, track change version).</u>

3. Perform experiments that would differentiate between the models (see above).
Show that inhibition of JAK-STAT and STAT pathways inhibit USP30-AS1 expression in the context of influenza infection

We appreciate the reviewer for bring up this important point. To address this, we performed CA04 infection while keeping JAK-STAT signaling ablated. It showed that CA04 infection in JAK-STAT signaling inhibited cells did not affect the activation of USP30-AS1 (Fig 4D). This might indicate the involvement of other immune signaling, potentially viral sensor signaling. Based on literature, viral sensor signaling also directly controls the activation of inflammatory cytokine genes. This is also supported by literature that viral recognition signaling can directly activate a range of pro-inflammatory genes upon viral RNA detection (Akira, Uematsu et al. 2006, Pichlmair and Reis e Sousa 2007, Iwasaki 2012). We believe results from this new experiment can provide additional insights to guide future work. <u>We have included this into our revised manuscript and commented on the new findings (Fig 4D and Lines 317-340 and Lines 498-509).</u>

Reviewer #3:

1. USP30-AS1 upregulation in IAV infected respiratory epithelium cultured at the air-liquid interface should be shown.

We thank the reviewer for the important suggestion. The time for preparing fully differentiate respiratory epithelial cell cultured at air-liquid interface is tedious and highly time-consuming process (~3-4 weeks). We therefore attempted to address this point using two different approaches:

1) We used primary human alveolar epithelial cells and infected these cells with multiple IAV subtypes. This experiment might in part mimic the suggested experiment. Our new experiments showed that the expression of USP30-AS1 was remarkably enhanced in infected primary cells compared to mock control, suggesting the robustness of the transcriptional activation of USP30-AS1 to different subtypes of IAV infection. We have included these new data in our revised manuscript (Fig 1B and Lines 190-194, track-change version).

2) A recent publication (Enguita, Leitao et al. 2022) showed that the detected expression of *USP30-AS1* was positively correlated with increased viral load in nasal swabs from Covid-19 patient, suggesting the expression of *USP30-AS1* might potentially correlated with some viral infections *in vivo*. We have cited this reference and commented on this finding (Ref #56 and Lines 534-538, track-change version).

2. The question how USP30-AS1 inhibits IAV infection remains inadequately addressed. Upregulation of pro-inflammatory genes should be confirmed on the protein level and it should be investigated how this upregulation impacts viral protein expression.

We appreciate the reviewer for mentioning the important point. We further validated the upregulation of multiple pro-inflammatory protein expression in supernatant of CA04 infected *USP30-AS1* knock-out cells compared to infected WT cells (i.e. the same experimental treatment used for bulk RNA-seq) by beads-based immunoassay. Our data confirm that deletion of *USP30-AS1* can trigger high inflammatory response in both RNA and protein level during IAV infection. We have included this new dataset in our revised version (Fig 5E, track-change version).

As to the question of how this upregulation impacts viral protein expression, we think that there are lots of possibilities from multiple facets, therefore it requires systematic work to address the question. We believe this is a big topic which is beyond the scope of our current study. We, however, fully acknowledge this and highlight this as a limitation of our study (Lines 527-529).

3. It should be investigated whether the impact of USP30-AS1 on inflammatory responses is IAV specific or also seen in the context of other respiratory infections.

We thank the reviewer for the constructive suggestion. The scope of our study is limited to influenza A virus infection only. Interestingly, a recent study on human respiratory samples suggest that the expression of USP30-AS1 can be stimulated by SARS-CoV-2. We have cited this recent study and commented on the role of USP30-AS1 in other respiratory virus infections (Ref #56 and Lines 534-540, track-change version).

Part III – Minor Issues: Editorial and Data Presentation Modifications
Please use this section for editorial suggestions as well as relatively minor modifications of existing data that would enhance clarity.

Reviewer #1:
1. The rationale to select USP30-AS1 over IRF1-AS1, also differentially expressed in infections with different influenza strains, should be provided.

We thank the reviewer for mentioning the important question. Our rationale for choosing *USP30-AS1* over *IRF1-AS1* is because there is limited work on *USP30-AS1* in the context of immune regulation or infection. Therefore, this work would be important to fill this knowledge gap. As for *IRF1-AS1*, there are some publications suggesting that it immunologically relevant to inflammation response and associated with the expression of *IRF1*, an important interferon regulatory transcription factor (Barriocanal, Prats-Mari et al. 2022, Johnson, Sargsyan et al. 2024). For us, the study of USP30-AS1, while more challenging to decipher, is clearly more innovative. We have added this rationale in the revised result and discussion sections (Lines 185-188 and Lines 462-465, track-change version).

2. Regarding the "replication" of the virus, these two statements might be apparently contradictory, please revise the wording: "USP30-AS1 had no significant effect on viral RNA transcription or replication" (lines 180-181)" and "the absence of USP30-AS1 may enhance the production of viral protein and promote virus replication". In this last sentence, "virus replication" might be replaced by "virus growth".

We thank the reviewer for noting the issue. We have replaced "virus replication" by "virus growth" or "virus propagation" as suggested (e.g. Lines 26 and 228, track-change version).

3. Table 1, which provides essential information about the cell lines used for the influenza infection experiments analyzed in the manuscript, should be moved from supplementary information to the main manuscript.

We thank the reviewer for bringing up this point. We have done this as recommended (Table 1, track-change version).

4. Fig. 3C is not mentioned in the manuscript. It could be kept in supplementary S5 Figure, since it is highly related to Fig.S5 G, which shows the expression of NP in USP30-AS1-/- cells infected with influenza virus.

We thank the reviewer for mentioning the issue. Accordingly, the revision has been made as suggested. These results are now presented in a single figure in the revised version (S6A and S6B, revised version).

Reviewer #2:
1. The figures and order of presentation are a bit confusing. Why is fold increase in expression after HK68 infection presented in the supplement and not part of Figure 2?

We thank the reviewer for mentioning the issue. We have made the presentation format more consistent as suggested. Please also see our reply to the same reviewer (Part I, Major Comment) for further explanation.

2. Similarly, what is the purpose of S5G? You have already shown this in Figure 3. Consider combining to enforce the point that protein expression is higher.

We thank the reviewer for mentioning the issue. Accordingly, the revision has been made as suggested. These results are now presented in a single figure in the revised version (S6A and S6B, revised version).

3. The role of S7 is unclear as it has limited relevance to this paper.

We thank the reviewer for noting the issue. We have deleted this figure as suggested.

Reviewer #3:

1. The authors should refer to influenza A viruses and not generally to influenza viruses.

We thank the reviewer for noting the issue. We have specified influenza A virus (IAV) throughout the manuscript.

References used in this reply

Akira, S., S. Uematsu and O. Takeuchi (2006). "Pathogen recognition and innate immunity." Cell **124**(4): 783-801.
Barriocanal, M., L. Prats-Mari, N. Razquin, C. Prior, J. P. Unfried and P. Fortes (2022). "ISR8/IRF1-AS1 Is Relevant for IFNalpha and NF-kappaB Responses." Front Immunol **13**: 829335.
Enguita, F. J., A. L. Leitao, J. T. McDonald, V. Zaksas, S. Das, D. Galeano, D. Taylor, E. S. Wurtele, A. Saravia-Butler, S. B. Baylin, R. Meller, D. M. Porterfield, D. C. Wallace, J. C. Schisler, C. E. Mason and A. Beheshti (2022). "The interplay between lncRNAs, RNA-binding proteins and viral genome during SARS-CoV-2 infection reveals strong connections with regulatory events involved in RNA metabolism and immune response." Theranostics **12**(8): 3946-3962.
Iwasaki, A. (2012). "A virological view of innate immune recognition." Annu Rev Microbiol **66**: 177-196.
Johnson, J. L., D. Sargsyan, E. M. Neiman, A. Hart, A. Stojmirovic, R. Kosoy, H. Irizar, M. Suarez-Farinas, W. M. Song, C. Argmann, S. Avey, L. Shmuel-Galia, T. Vierbuchen, G. Bongers, Y. Sun, L. Edelstein, J. Perrigoue, J. E. Towne, A. O. Hall, K. A. Fitzgerald and K. Hoebe (2024). "Gene coexpression networks reveal a broad role for lncRNAs in inflammatory bowel disease." JCI Insight **9**(3).
Pichlmair, A. and C. Reis e Sousa (2007). "Innate recognition of viruses." Immunity **27**(3): 370-383.
